# Grandmother presence improved grandchild survival against childhood infections but not vaccination coverage in historical Finns

**DOI:** 10.1098/rspb.2023.0690

**Published:** 2023-05-31

**Authors:** Susanna Ukonaho, Simon N. Chapman, Michael Briga, Virpi Lummaa

**Affiliations:** ^1^ Department of Biology, University of Turku, 20014 Turku, Finland; ^2^ INVEST Flagship Research Centre, University of Turku, 20014 Turku, Finland; ^3^ Infectious Disease Epidemiology Group, Max Planck Institute for Infection Biology, 10117 Berlin, Germany

**Keywords:** grandmothers, mortality, smallpox, measles, vaccination

## Abstract

Grandmother presence can improve the number and survival of their grandchildren, but what grandmothers protect against and how they achieve it remains poorly known. Before modern medical care, infections were leading causes of childhood mortality, alleviated from the nineteenth century onwards by vaccinations, among other things. Here, we combine two individual-based datasets on the genealogy, cause-specific mortality and vaccination status of eighteenth- and nineteenth-century Finns to investigate two questions. First, we tested whether there were cause-specific benefits of grandmother presence on grandchild survival from highly lethal infections (smallpox, measles, pulmonary and diarrhoeal infections) and/or accidents. We show that grandmothers decreased all-cause mortality, an effect which was mediated through smallpox, pulmonary and diarrhoeal infections, but not via measles or accidents. Second, since grandmothers have been suggested to increase vaccination coverage, we tested whether the grandmother effect on smallpox survival was mediated through increased or earlier vaccination, but we found no evidence for such effects. Our findings that the beneficial effects of grandmothers are in part driven by increased survival from some (but not all) childhood infections, and are not mediated via vaccination, have implications for public health, societal development and human life-history evolution.

## 1. Introduction

In many species, kin help raise young after birth or hatching [1–4], and this support is an important determinant of offspring survival and reproduction [2,5–8]. For example, in several historical and contemporary human societies, grandmothers contribute to raising grandchildren, thereby improving grandchild survival as well as their birth rate [2,5,9]. These contributions can have important evolutionary consequences through increasing the fitness of offspring and grandchildren [10], and hence increasing the women's own fitness indirectly. According to the grandmother hypothesis, such fitness benefits have been important in the evolution of post-reproductive lifespan in humans and other species [11].

While the contribution of grandmothers to the improvement of grandchild survival is well established in several societies [2,12–18] (but see [19–23]), the mechanisms underlying this process remain less clear. For example, in hunter–gatherer societies, grandmothers improved the survival of grandchildren through increased food provisioning [24]. Another way that grandmothers could provide fitness benefits is by providing knowledge in childcare [25–27], particularly during critical times such as sickness and epidemics. In historical and in several contemporary societies, childhood infectious diseases were and are leading causes of death in children under the age of 5 years [28–32]. To the best of our knowledge, whether grandmother presence improves grandchild survival from childhood infections in historical societies remains unknown.

High smallpox mortality decreased rapidly in many countries after the introduction of the smallpox vaccine in the early 1800s [29]—the only vaccine available until the twentieth century. One way that grandmothers could improve the survival of grandchildren is through encouraging the uptake and timing (i.e. earlier) of vaccination against childhood infections. Indeed, in contemporary societies, the grandmother's opinion has been listed as one of the top factors contributing to parents' attitudes towards vaccines [33]. For example, in twentieth-century Lebanon, grandmothers and great-grandmothers were considered the second most important influence on child health issues after healthcare providers [34], and high vaccine-positivity among Indian grandparents in the UK towards the measles, mumps and rubella (MMR) vaccine correlated with high coverage among their grandchildren [35].

In this study, we investigate the effect of grandmother presence on all-cause and cause-specific mortality of grandchildren, and whether grandmother presence promotes grandchild vaccination. First, we use a 140-year-dataset from historical Finland (1761–1900) to test whether the previously described beneficial effects of grandmothers on grandchild survival in this population [5,18] are mediated via improved survival from the childhood infections smallpox and measles, via pulmonary infections, diarrhoea and/or via avoiding accidental deaths. Second, if there are any beneficial effects of grandmothers on childhood survival from smallpox, these effects could be mediated via increased vaccination coverage. To test this hypothesis, we used a recently compiled 30-year longitudinal dataset on vaccination coverage in rural nineteenth-century Finnish families [36] to test whether grandmother presence (i) increased vaccination coverage and (ii) reduced the age at vaccination.

## 2. Methods

### (a) Data

For the purpose of this study, we combined two datasets: (i) a large multigenerational demographic dataset of pre-industrial Finnish families [18] and (ii) vaccination records manually digitized from church records held in Finnish national and provincial archives [36]. The large multigenerational dataset of pre-industrial Finnish families was previously used to identify the beneficial effects associated with grandmother presence on grandchild survival [5,18]. In brief, these data were by law required to be collected by local clergymen from 1749 onwards [37], and we have digitized individual records on, for example, the birth, marriage and death dates and the cause of death of the local inhabitants of twelve parishes across Finland (Ikaalinen, Jämijärvi, Honkajoki, Tyrvää, Rymättylä, Karvia, Kustavi, Parkano, Hiittinen, Pulkkila, Rautu, Jaakkima). We confined our study period to birth years 1761–1900, as we had little data before 1761 and because after 1900 smallpox mortality was negligible, we had no vaccination data for the period and wanted to avoid confounding effects from other public health services that were established throughout the twentieth century [38]. Additionally, childhood mortality declined greatly after the turn of the century [39], so the need for grandmother help was also greatly reduced. To monitor childhood survival, we included all individuals monitored from birth until the age of 15 years on 9705 individuals resulting in 81 019 person-years, of which 3857 individuals died before the age 15. For the vaccination coverage data, we collected and digitized two parishes (Ikaalinen and Jämijärvi) between 1870 and 1900, which resulted in a total of 1594 children between 0 and 15 years of age. Most individuals were vaccinated within the first 2 or 3 years after birth, with no records of vaccinations of children older than 8 years old. For further details on Finland's vaccination campaign against smallpox, see Ukonaho *et al*. [36]. In brief, the vaccination campaign started in Finland in 1802, but consistent collection of vaccination records with details on the vaccinated individuals became more common around the 1840s, depending on the parish. Although the vaccination campaign faced high hesitancy, the vaccination coverage of children was on average 80% (interannual SD: 22%) during our study period [36]. However, large smallpox epidemics occurred until the mandatory vaccination law was introduced in 1883 after which smallpox prevalence decreased until the last Finnish case was diagnosed in 1941.

To study the cause-specific effects of grandmothers on grandchild mortality, we identified, based on the historical records, five causes of death for our study: smallpox, measles, pulmonary infections, diarrhoeal deaths and accidents. These were among the most common causes of death for children under the age of 15, capturing nearly half of all 3857 deaths during our study period, and among the easiest to identify by the local priests who had little to no medical training (electronic supplementary material, table S1). Smallpox and measles are both lethal childhood infections transmitted via droplets and for which the various skin rashes made identification easy [29]. In the eighteenth and early nineteenth centuries, medical care was close to non-existent in Finland, which meant that sick children were cared for at home [38]. Those who had been infected and survived gained lifelong immunity in both cases. For further details on smallpox and measles in historical Finland, see studies by Ketola *et al*. [31] and Briga *et al*. [40], and see also electronic supplementary material, table S1. In our study, ‘pulmonary infections' is an umbrella term for the causes of death that were registered as pneumonia, tuberculosis and ‘unknown respiratory infections'. We chose this umbrella approach because in historical Finland causes of death were not recorded by medical practitioners but by the local priests and clergymen and hence we took the conservative approach assuming that it is difficult to consistently distinguish between different pulmonary infections by different clergymen across parishes and across time. Similarly, many infections, including cholera, dysentery and typhoid fever, caused diarrhoea in children, and generally these could not be distinguished in eighteenth- and nineteenth-century Finland. Hence, we grouped deaths that in the historical records were identified as ‘diarrhoea' as one cause. The category of accidental deaths also included many causes, including drowning, suffocation, falls, burned in a fire, frozen in cold weather and kicked by a horse.

### (b) Statistical analyses

To identify the association between grandmother presence and grandchild mortality, we performed survival analyses using the counting process formulation of the Cox proportional hazard (CPH) model [41–43], which we performed in R version 3.6.1 [44] with the function *coxme* in the R package *survival* following Therneau *et al*. [45,46]. All models included maternal grandmother presence, which we categorized as a two-level variable (0−absent, 1−present) based on whether the grandmother had been confirmed alive or not. The counting process allows the coefficient to be estimated at each time point and thus time-dependent changes in grandmother status can be accounted for. We used data at the annual level and hence we included one row per individual-year until either death or the age of 15 years, after which all individuals were censored. To test the effect of grandmother presence on multiple causes of death, we performed a competing risk analysis [46]. In brief, multiple causes of death are included in one model, and any cause of death can occur at each individual-year until death or censoring. Models fulfilled all CPH assumptions as checked using scaled deviance and martingale residual plots [41,43].

In survival analysis models, we first included paternal grandmother presence in maternal grandmother models. We confirmed the maternal grandmother effect, but observed no paternal grandmother effect, indicating that grandchild survival benefits are driven by the maternal but not by the paternal grandmothers, a result which was consistent with previous results [18]. Hence, we focused on maternal grandmother effects for the rest of our study. Grandmothers living closer are most likely to affect grandchild survival [47]. For the all-cause and cause-specific analyses, we lacked the power to distinguish between grandmothers living in the same or in different parishes as their grandchildren and we therefore chose the conservative approach to include the grandmothers as ‘alive' independently of the distance she lived from her grandchildren. For the vaccination analyses, we were able to make this distinction and performed two analyses, one only with grandmothers whose parish of residence was known and a second including grandmothers with unknown residence. Both approaches gave consistent results and here we show the analyses including grandmothers without known parish of residence.

Previous research in pre-industrial Finland has found differences in female life-history traits and life-course events between social classes [48,49], and therefore it is important to account for it in models relating to life-history (here, grandchild survival). To avoid confounding grandmother survival and social class, we included childhood social class in all models, which we found to correlate significantly with grandmother presence (electronic supplementary material, table S2). We classified social class as a 3-level variable, with class 1 being wealthy landowners, class 2 tenant farmers and small-occupation holders with average income, and class 3 poor landless [49]. Since there was a possibility (albeit small) of social class mobility from childhood to adulthood, childhood social class was determined by taking the social class of the father; women rarely had occupations of their own, and their social class was typically determined by the occupation of their husband [49]. It has been shown that grandmothers increase the number of grandchildren in a family [5], which in turn can affect disease transmission and/or accidents. To avoid confounding the number of grandchildren with grandmother effects, we also included the number of siblings as a covariate in all models. In all models, we included birth parish and birth cohort as a random intercept, to account for spatial and temporal clustering. In addition, in the survival models we also nested mother ID within birth parish to account for multiple children coming from the same families.

To investigate whether increased survival against childhood infectious diseases was mediated by vaccination, we tested the effect of grandmother presence on the vaccination coverage (binary response variable: 0 = non-vaccinated, 1 = vaccinated) and whether grandmothers decreased the age at vaccination. We performed the vaccination coverage analyses using binomial generalized mixed models with the R package *lme4* [50] and the age-at-vaccination analysis using the aforementioned cox proportional hazards models (see above). All model residuals fulfilled the model assumptions, which we checked with the function ‘simulateResiduals' of the R package ‘DHARMa' [51]. We quantified statistical ‘significance' of predictor variables using *p*-values and with the model selection approach based on the second-order Akaike information criterion (AICc) [52,53], with the function *AICc* of the R package ‘MuMIn' [54], which gave consistent results.

## 3. Results

**Figure 1.**
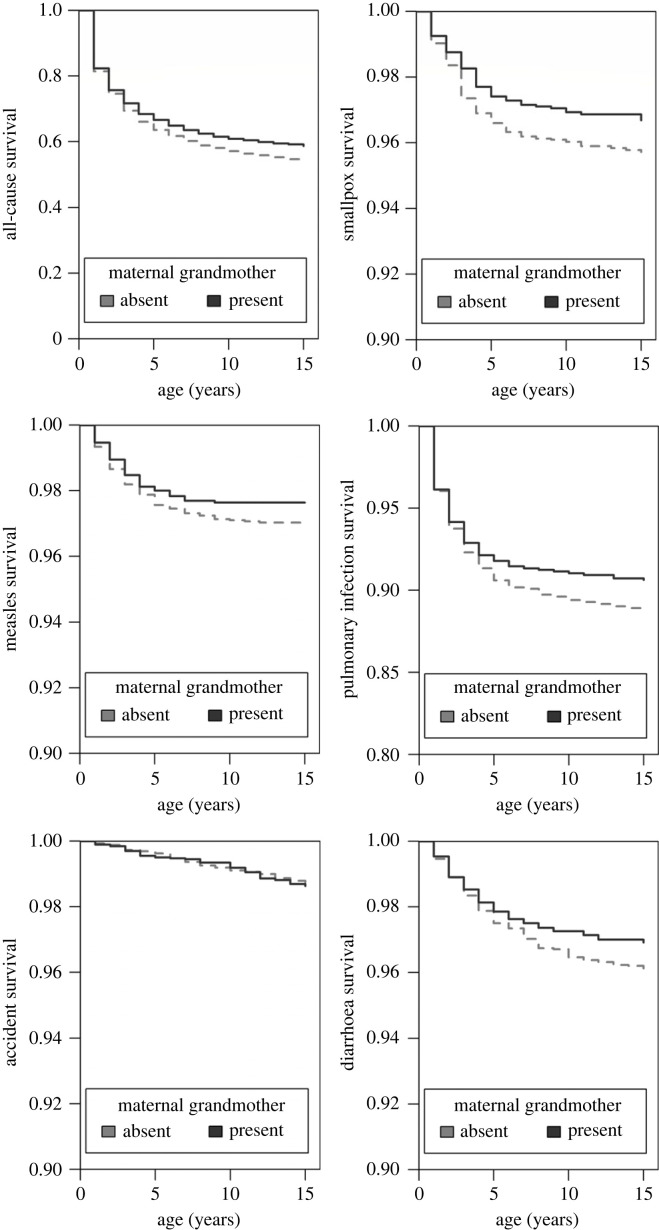
Maternal grandmothers prevented all-cause and cause-specific mortality from smallpox, pulmonary infections and diarrhoea in children age 0–15 during the eighteenth and nineteenth centuries.

Overall, during the study period, 40% of children died before the age of 15 years and 56% had their grandmother alive during at least a part of their childhood. First, we confirmed the results from previous studies in the same population which showed that grandmothers improved all-cause survival of their grandchildren. Indeed, on average, between the age of 0−15 years, individuals with maternal grandmothers had a mortality ratio that was 20% lower relative to individuals without grandmothers (s.e.: 4%; figure 1 and table 1*a*), which by age 15 years translated into a 4.4% difference in survival (figure 1).

**Table 1.** Model outputs for grandmother effects on grandchild mortality for (*a*) all-cause mortality, (*b*) cause-specific mortality, (*c*) vaccination coverage and (*d*) age at vaccination. Models (*a*) and (*d*) are time-dependent mixed-effects Cox proportional hazards, (*b*) a competing-risk mixed-effect Cox proportional hazard and (*c*) a generalized mixed-effect model. The table shows model estimate, hazard ratio, standard error, *t*- or *z*-value and *p*-value.

dependent variable	estimate	hazard	s.e.	*t*-value	*p*-value
(*a*) all-cause mortality					
MGM_present_	−0.23	0.79	0.04	−5.96	<0.001
living siblings	−0.21	0.81	0.01	15.05	<0.001
socio-economic class_2_	0.09	1.09	0.05	1.69	0.09
socio-economic class_3_	0.11	1.11	0.06	1.84	0.07
(*b*) smallpox mortality					
MGM_present_	−0.36	0.70	0.13	−2.68	<0.01
living siblings	−0.19	0.83	0.05	−3.88	<0.001
socio-economic class_2_	−0.07	0.94	0.16	−0.40	0.69
socio-economic class_3_	0.00	1.00	0.19	−0.02	0.98
measles mortality					
MGM_present_	−0.18	0.84	0.16	−1.11	0.27
living siblings	−0.00	1.00	0.06	−0.05	0.96
socio-economic class_2_	0.01	1.01	0.20	0.05	0.96
socio-economic class_3_	0.01	1.01	0.22	0.05	0.96
pulmonary mortality					
MGM_present_	−0.20	0.82	0.08	−2.67	<0.01
living siblings	−0.13	0.88	0.03	−4.81	<0.001
socio-economic class_2_	−0.03	0.98	0.09	−0.27	0.79
socio-economic class_3_	0.17	1.18	0.10	1.60	0.11
accident mortality					
MGM_present_	0.08	1.08	0.20	0.40	0.69
living siblings	−0.13	0.88	0.07	−1.88	0.06
socio-economic class_2_	0.69	1.99	0.23	2.97	<0.01
socio-economic class_3_	0.88	2.40	0.27	3.28	<0.001
diarrhoea mortality					
MGM_present_	−0.51	0.60	0.18	−2.83	<0.01
living siblings	−0.41	0.67	0.06	−6.50	<0.001
socio-economic class_2_	0.27	1.31	0.27	0.99	0.32
socio-economic class_3_	−0.26	0.77	0.33	−0.79	0.43
(*c*) vaccination coverage					
	estimate	hazard	s.e.	*z*-value	*p*-value
intercept	3.80	n.a.	1.97	1.93	0.05
MGM_present_	0.02	n.a.	0.16	0.12	0.91
socio-economic class_1_	0.73	n.a.	0.24	2.97	<0.01
socio-economic class_2_	0.16	n.a.	0.18	0.88	0.38
(*d*) age at vaccination					
	estimate	hazard	s.e.	*t*-value	*p*-value
MGM_present_	0.10	1.11	0.05	1.88	0.06
socio-economic class_1_	0.17	1.18	0.08	2.13	0.03
socio-economic class_2_	−0.07	0.93	0.07	−1.05	0.29

Next, we quantified whether the benefits of maternal grandmother presence on grandchild survival were cause-specific. Present maternal grandmothers were associated with a reduction in hazard ratio from smallpox, pulmonary infections and diarrhoea of children age 0−15 years by 20−40% compared to children without maternal grandmothers (HR_sp_ = 0.70, s.e._sp_ = 0.13, *p*_sp_ < 0.01; HR_pul_ = 0.82, s.e._pul_ = 0.08, *p*_pul_ < 0.01; HR_dia_ = 0.60, s.e._dia_ = 0.18, *p*_dia_ < 0.01; figure 1 and table 1*b*), which by age 15 years translated into increases in survival of respectively 1.0%, 1.9% and 0.8% for smallpox, pulmonary infections and diarrhoea (figure 1). Children with present maternal grandmothers also had reduced measles mortality but the difference was not statistically significant (HR = 0.84, s.e. = 0.16, *p* = 0.27, table 1*b*). By contrast, maternal grandmothers did not affect the mortality due to accidents (HR = 1.08, s.e. = 0.20, *p* = 0.69, table 1*b*).

For all causes of death, except for measles and accidents (HR_mea_ = 1.00, s.e._mea_ = 0.06, *p*_mea_ = 0.96; HR_acc_ = 0.88, s.e._acc_ = 0.07, *p*_acc_ = 0.06), there was a positive association between survival and the number of living siblings (HR = 0.67–0.88, s.e. = 0.03–0.06, table 1*b*). There was no association between socio-economic status and survival from infections, but children from higher socio-economic status were less likely to die from accidents (table 1*b*).

**Figure 2.**
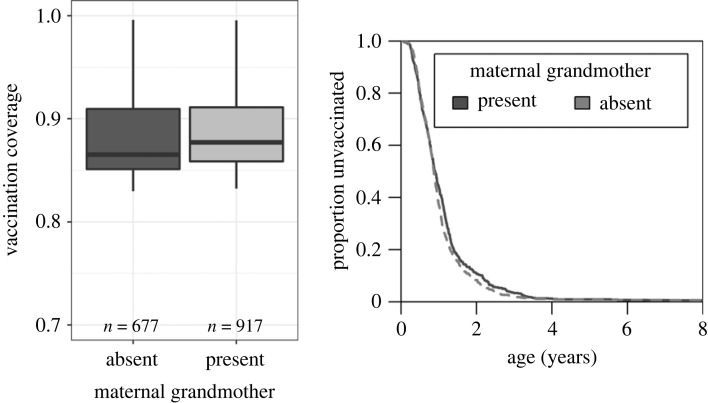
Maternal grandmother presence did not have an effect on vaccination coverage for children age 0–15 in southwest Finland. Grandchildren got vaccinated later when their maternal grandmothers were nearby and alive.

During the period 1870–1900, 88% of the children in our sample got vaccinated against smallpox, at an average age of 1 year. We observed no differences in smallpox vaccination coverage between children with or without maternal grandmothers (*z* = 0.12, *p* = 0.91; table 1*c* and figure 2). It is possible that children with grandmothers get vaccinated earlier than children without, which can confer protection against smallpox at an earlier age. However, if anything, we found a weak statistical support for the opposite result, namely that children with a grandmother were vaccinated on average just over one month later than children without a grandmother (s.e.: 38 days, *z* = 1.88, *p* = 0.06; table 1*d* and figure 2). Our study period overlaps with the introduction of a mandatory smallpox vaccination law (year 1883), which could diminish an effect of grandmothers on vaccination. Hence, we repeated the analysis including before versus after the vaccination law as a factor in interaction with grandmother presence, but we found no support for such change (*β*_interaction_ = 0.17, s.e. = 0.41, *p* = 0.67).

## 4. Discussion

To identify possible ways that grandmothers benefit grandchild survival, we used extensive historical data from Finland that included causes of death, vaccination records against smallpox and family configuration to quantify the effects of grandmothers on cause-specific mortality and vaccination. Childhood infections were a leading cause of early mortality in eighteenth- and nineteenth-century Finland [31,32,55]. Maternal grandmothers improved all-cause survival of their grandchildren, which was at least in part mediated by a decrease in mortality due to smallpox, pulmonary infections and diarrhoea. The increased survival was not mediated via vaccination, since grandmother presence did not alter the coverage of the smallpox vaccine, the only vaccine that existed at that time. Here, we discuss the implications of our results for human evolutionary biology and for public health interventions.

We found that maternal but not paternal grandmothers improved overall survival of their grandchildren, which supports the findings of earlier studies on this population [18,56] and elsewhere [2]. Studies in some other populations did not find beneficial effects of maternal grandmothers [2], although some of these studies did not distinguish between maternal and paternal grandmothers [19,23]. In our historical study population, childhood mortality was high and infectious diseases were the leading causes of death [32] (electronic supplementary material, table S1), which may increase the benefit of grandmother help on grandchild survival. In our study, paternal grandmother presence did not improve grandchild survival. Grandchildren often resided in the same household with their paternal grandmother, while maternal grandmothers typically lived close by but not under the same roof. Especially in patrilocal populations, grandchildren could even compete for resources with their paternal grandmothers, leading to an adverse effect on grandchild survival [56–59]. Hence, our results are consistent with previous studies.

Grandmother presence increased survival from smallpox, pulmonary infections and diarrhoea, but not from measles or accidents. This could be caused by several possible mechanisms. Grandmothers can help with childcare, food provisioning and/or the transmission of knowledge or even provide monetary investments [60]. The early recognition of symptoms can be important, which together with improved hygiene or access to water and food, is important in the early care for diarrhoea [61]. Indeed, data from the early twentieth century indicate that improvements in the quality of life, hygiene and/or food abundance were important in decreasing mortality from childhood infections long before the use of antibiotics or the common roll-out of vaccines [55,62]. Historical incidence data are extremely rare, but ideally one would test whether grandmothers decrease case fatality rates (i.e. the proportion of diagnosed people who die from a specific cause from childhood infection in grandchildren would be useful).

Alternatively, grandmothers could help with disease prevention, thereby decreasing the probability that grandchildren contracted certain infections. While this is possible for some infections and could explain some infection-specific results, we consider it unlikely for infections like smallpox, which was abundantly present in eighteenth- and nineteenth-century Europe [63–65]. An alternative explanation could be genetic differences as descendants from long-lived grandmothers, who survived smallpox and other illnesses, would also likely share similar genetic makeup. However, we believe such genetic differences to be rather unlikely as only maternal but not paternal grandmothers are associated with the survival of grandchildren. The grandmother effect is also unlikely a result from geographical or temporal variation, which we control for in our models.

For measles, the results are in the same direction as for other infections, but less pronounced and not statistically significant (table 1*b*). We believe this may be caused by two reasons. First, it is possible that grandmothers were less effective at protecting against measles relative to other infections because measles has a distinct immunosuppressive mechanism: measles virus destroys B-cells and as a consequence, the host loses part of its immunological memory [66], which leaves children more at risk of dying from measles and other infections [67,68]. If grandmothers help their grandchildren primarily through providing nutrition and childcare, these measures will likely be less effective when there is an inevitable loss of immunological function [69]. Second, because of the immunosuppressive mechanism, children infected with measles are also more likely to die from other infections, but these would not be reported as measles deaths. Hence, measles-related deaths can be underreported thereby weakening the impact of grandmother effects.

We expected grandmother care or wisdom to prevent accidental deaths, such as deaths from drowning, suffocation or freezing, but the negative result (table 1*b*) is contrary to our expectation. On the other hand, preventing accidental deaths, especially drownings, which encompassed over 50% of all accidental deaths in our study areas situated near lakes or within the archipelago off the Finnish coastline, is difficult even in contemporary societies [70,71].

Our study shows that the benefits of grandmother presence was not mediated via increased vaccination coverage, since we found no evidence that children with a living grandmother had a higher vaccination coverage or earlier age at vaccination. This is in contrast with some studies from contemporary populations which have shown that grandmothers promote vaccination [34,35,72]. Historical and contemporary populations differ in many ways and the incentives for or against vaccination may be very different in historical versus contemporary populations. For example, the smallpox vaccine was the first vaccine ever produced [73], and this historical change is likely to be linked with serious concerns about vaccine safety and efficacy; grandmothers in eighteenth- and nineteenth-century Finland are unlikely to have experienced similar benefits of vaccination campaigns or public health developments in general relative to grandmothers during the twentieth century [62,74–76]. On the other hand, before the development of the smallpox vaccine, smallpox was the leading cause of mortality among children aged 5 years or younger and smallpox caused many outbreaks in Finland during the eighteenth and nineteenth centuries [29]. Hence, most grandmothers likely had first-hand experiences with childhood infections, either themselves, via friends and/or own children succumbing to this disease, which can be a strong incentive to value any means to avoid further casualties. Grandmother care changed over time, and grandmothers could have had a more important role in getting their grandchildren vaccinated at the beginning of the vaccination campaign (in the early 1800s), rather than near the end of nineteenth century when vaccination became mandatory. Our vaccination data do not cover the early 1800s, but note that we observed no difference in grandmother effect on vaccination coverage before versus after the law.

In conclusion, we found that grandmother presence contributes to the survival and reproductive success of kin by increasing survival from childhood infections. Consistent with the idea that intergenerational transmission of knowledge is important in grandmothering in humans and other species [8,60,77,78], our study indicates a significant role of grandmother care. However, it is surprising that grandmothers neither affect vaccination coverage nor accidental deaths. Therefore, grandmother help is likely to be more intricate than previously assumed, and this is important both for public health and evolutionary biology. For example, grandmothers could be less likely to recommend vaccines they did not have themselves, but more likely to recommend vaccines they had experience with (e.g. MMR vaccine [35]), and this warrants further study.

## Data Availability

All church book records can be accessed via the Genealogical Society of Finland: https://hiski.genealogia.fi/hiski/oc13w?en. The data are provided in electronic supplementary material [79].
